# Resveratrol and Omega-3 Fatty Acid: Its Implications in Cardiovascular Diseases

**DOI:** 10.3389/fcvm.2015.00038

**Published:** 2015-12-11

**Authors:** Bibhuti Bhusan Kakoti, Diana G. Hernandez-Ontiveros, Manjir Sarma Kataki, Kajri Shah, Yashwant Pathak, Siva Kumar Panguluri

**Affiliations:** ^1^Department of Pharmaceutical Sciences, Dibrugarh University, Assam, India; ^2^College of Pharmacy, University of South Florida, Tampa, FL, USA

**Keywords:** resveratrol, omega-3 fatty acid, oxidative stress, endoplasmic reticulum stress, cardiovascular diseases, coronary artery diseases

## Abstract

The present review aims at summarizing the major therapeutic roles of resveratrol and omega-3 fatty acids (O3FAs) along with their related pathways. This article reviews some of the key studies involving the health benefits of resveratrol and O3FAs. Oxidative stress has been considered as one of the most important pathophysiological factors associated with various cardiovascular disease conditions. Resveratrol, with the potent antioxidant and free radical scavenging properties, has been proven to be a significantly protective compound in restoring the normal cardiac health. A plethora of research also demonstrated the reduction of the risk of coronary heart disease, hypertension, and stroke, and their complications by O3FAs derived from fish and fish oils. This review describes the potential cardioprotective role of resveratrol and O3FAs in ameliorating the endoplasmic reticulum stress.

## Introduction

Cardiovascular complications continue to be among the list of leading causes of death worldwide. According to centers for disease control and prevention (CDC) reports, more than half a million people in the USA die of heart disease every year, one in every four deaths every year (1). Increasing evidences suggest that metabolic syndrome constitutes a significant risk for cardiovascular diseases, which can also cause type 2 diabetes, obesity, and oxidative stress (2, 3). Effective tactics to reduce these conditions include immediate weight loss (4), and long-term dietary strategies enhancing health by maintaining the body weight (5). There are multiple non-pharmacological approaches, such as alternative medicine, that have the potential to reduce cardiovascular risks; resveratrol and omega-3 fatty acid (O3FA) are two well-known natural products that come under this category.

Resveratrol is chemically *trans*-3,5,4′-trihydroxystilbene. It is a polyphenol phytoalexin compound naturally abundant in a variety of plant species, including white hellebore (*Veratrum grandiflorum O. Loes*), Japanese knotweed (*Polygonum cuspidatum*), grapes, blueberries, peanuts, wine, and mulberries (6, 7). Resveratrol came into the limelight following the discovery of the cardioprotective nature of red wine (8). This has paved the way for new research activities involving clinical health benefits of resveratrol. A growing body of research therefore indicated that resveratrol could improve a wide variety of disease conditions, including cardiovascular diseases, cancer, ischemic disorders, and neurodegenerative disorders (9). It is also postulated that resveratrol can increase the stress resistance and also slow down the aging process to increase the life span in diverse organisms from microorganisms to vertebrates (6).

Resveratrol is one of the well-known natural compounds, which is well-studied for its free radical scavenger and antioxidant properties. It can modulate various intracellular signal transduction pathways and demonstrate therapeutic effects, including cell survival, modulation of apoptosis, and angiogenesis (10, 11). Growing evidences suggests the anti-cancer activity of resveratrol and its potential as a therapeutic as well as a chemo-preventive agent (12). Scientific outcomes demonstrated the potential use of resveratrol in various cancers (13–25). However, pre-clinical results were not well reflected in clinical studies in human. It may be due to the pharmacokinetic issues pertaining to resveratrol. It is lipophilic in nature and has a short half-life as well.

The use of O3FAs as a widespread alternative has been practiced globally by many societies. O3FAs are essential for normal growth and development of the nervous system. The benefits of O3FA have been documented in the past 35 years (26–28). All these research data strongly support the use of O3FAs in reducing the risk of cardiac diseases.

The purpose of this review is to summarize the major antioxidant role of resveratrol and O3FA along with their related pathways and highlight promising therapeutic approaches. The benefits of combinational therapy with these two natural compounds were also discussed.

## Resveratrol and the Endoplasmic Reticulum Stress

The endoplasmic reticulum (ER) is a significant but complex intracellular organelle in the physiological system. It is a vital organelle involved in modification and synthesis of proteins, translational processes, and fabrication at the post-translational level. Physiologically, the integrity of ER is maintained for proper cellular functioning. Any stimuli from diverse origin can influence the ER environment and generate disturbance in terms of nutritional deficiency leading to a compromised pathological status. The stimuli can be of physiological and biochemical origin, which can produce moderate-to-severe abnormalities, including alterations in glycosylation, depletion of calcium stores, oxidative stress conditions, DNA damage, and other pathological conditions. These alternations generally produce a severe pathological stress condition in the ER known as ER stress or ER oxidative stress. This pathological stress is coexisted with the abnormal protein accumulations in the ER.

Many reports from animal and human studies have shown that oxygen deprivation in the heart causes ER stress of myocytes by accumulating unfolded and misfolded proteins, thereby triggering apoptosis (29–32). Also, the research data showed that ER stress may play a crucial role in the elevation of atherosclerotic plaque (33–35). Furthermore, recent evidences also suggest that a transcriptional factor, nuclear factor-E2-related factor (Nrf2), may play a central role to compensate ER stress conditions (36). This Nrf2 signaling has shown to upregulate the expression of proteasomal catalytic sub-units in several cell types, thereby contributing to the ER stress response by enhancing proteasomal-mediated ER-associated degradation (37). Other studies also suggest that interventions against ER stress and activation of Nrf2 significantly reduce myocardial infarct size in animals exposed to I/R injury and also reduce cardiac hypertrophy in heart failure animal models (38).

## Resveratrol as a Cardioprotective Agent

Resveratrol has shown beneficial and protective effects against most degenerative and cardiovascular diseases, including ischemia/reperfusion injury, atherosclerosis, hypertension, and heart failure. The adaptive response through the preconditioning-like action of resveratrol is linked with the cardioprotective effects of the compound. With the expression of cardioprotective genes and proteins, such as heat shock and antioxidant proteins, resveratrol demonstrated an adaptive stress response as the preconditioning (39). These effects of resveratrol have been correlated with the modulation of mTOR-Rictor survival pathways (40). Results of several *in vitro* and *in vivo* studies of resveratrol in animal models suggested LDL oxidation inhibition, suppression of aggregation of platelets, and reduction of ischemia–reperfusion-induced myocardial injury, which in turn indicated the promising cardiovascular protective effects of resveratrol (41–43). Resveratrol also revealed anti-inflammatory activity, which was postulated to be linked with nitric oxide (NO) production. This study also demonstrated cardioprotective action of resveratrol by inhibiting toll-like receptor 4 (TLR4)/NF-κB signaling pathway in a rat model of myocardial ischemia and reperfusion (MI/R) (44). Another very common cardiovascular disease is stroke, which is one of the main causative factors of deaths throughout the world population. Scientific investigations suggest oxidative stress as the predominant pathophysiological modality associated with stroke (45, 46). Recent data also suggest that the ability of resveratrol in ameliorating the oxidative stress conditions might be due to the aggravation of NO production along with its anti-inflammatory activity (47). Studies also suggested several mechanisms of action for resveratrol to justify its cardioprotective actions (48, 49). Resveratrol was also found to be protective against the cardio toxicity induced by the potent anti-cancer agent doxorubicin (50). These authors showed that resveratrol restored the mitochondrial structure as evident from the electron microscope examinations. A recent pre-clinical study also suggested that resveratrol alleviate the cardio toxicity induced by doxorubicin (51).

## Role of Omega-3 Fatty Acids as Antioxidant Agents in Cardiovascular Diseases

Two well-known fish/fish oil-derived O3FA are the eicosapentanoic acid (EPA) and docosahexanoic acid (DHA). DHA and EPA are vital nutrients required for brain and retina growth in order to have normal neuronal functioning and vision. These two in turn help humans develop their learning and mental abilities throughout life. These fatty acids are by-products derived from the metabolic conversion of the amino acid alanine (ALA) in the liver (52). Yet, human intake of O3FA is limited; they are not abundant in vegetables and grain food sources, and thus, they have to be supplemented directly. In addition, the metabolic change of alanine (ALA) to DHA/EPA in the body is limited; therefore, it is recommended to have sufficient amounts of fish, or O3FA directly as a dietary supplement.

The American Heart Association recommends 130 mg/day of DHA/EPA (combined), for people suffering from coronary heart disease. Various studies have indicated the benefits of fish consumption containing the O3FA, EPA/DHA, and lowering the risk of cardiovascular conditions, such as fatal heart attack, high cholesterol, and stroke (53–57). Yet, the signal transduction pathways and mechanisms associated with it and how they function have not fully been revealed.

## Mechanisms Associated with O3FA

Omega-3 fatty acids seem to have versatile properties with no significant drug interactions. The suggested mechanisms associated with the benefits of O3FA are stabilization of plaque formation, improving lipid profile, normalizing blood pressure, and anti-inflammatory and anti-arrhythmic properties (58). Nevertheless, there is still a major controversy over O3FA having benefits against arrhythmias, which was suggested by the Italian GISSI-Prevenzione study (59). The exact mechanism of its action in reducing cardiovascular risk is yet to be understood.

## O3FA and Oxidative Stress

One of the valuable properties of O3FAs is their antioxidative action. They seem to induce cardioprotection by ameliorating oxidative stress. Oxidative stress causes damage by the production of radicals that distress normal metabolic cell functioning by destroying lipids, proteins, and nucleic acids. Radical accumulation eventually causes structural cell membrane damage, vascular endothelial dysfunction, and cell death, often leading to cardiovascular diseases (60). A recent study in rats evaluated the combined effects of intermittent hypobaric hypoxia (IH) and O3FA on cardiac function, oxidative balance, and inflammatory state. Their findings showed that animals under IH have improved left ventricular function, reduced oxidative stress, and increased antioxidant enzyme expression when compared to the normobaric normoxia group. Supplementation of O3FA showed similar results to that of the IH group, suggesting that both conditions, IH and O3FA, induce functional improvement by antioxidant and anti-inflammatory mechanisms, thereby establishing cardioprotection (61).

Furthermore, another protective action of O3FA is to reinforce the body’s antioxidant defenses to improve cardiac function. Recent data suggest that DHA and EPA regulate cell membrane fluidity, organization, and permeability that affect signaling pathways and diffusion processes, with positive effects on key cardiovascular pathways (62). In addition, O3FAs manage to exert anti-inflammatory properties by controlling the synthesis of immune mediators (e.g., thromboxanes, prostaglandins, and leukotrienes) in murine cell lines and human primary monocyte-derived macrophages (63–65). These mediator molecules change the arachidonic acid metabolic pathway of prostaglandin E2 (PGE2), via the activation of cytosolic phospholipase A2 (cPLA2), cyclooxygenase 2 (COX-2), and the production of PGE2, as a cPLA2 inhibitor, and reduce other pro-inflammatory molecules in the heart (e.g., IL-1 and IL-6) (63). Also, a high O3FA enriched diet has shown benefits in a rat model with cardiac ischemia–reperfusion injury (66).

Furthermore, specific types of O3FAs can affect oxidative stress due to disparities in their chemical predisposition to oxidation. Experiments by Bellido et al. (67) showed an increase in activation of a redox-sensitive transcriptional factor, NF-κB, in peripheral blood mononuclear cells following a meal high in butter or walnuts but not olive oil (67). These experiments are interesting and require further expansion to determine specific fatty acid effects, and any other meals specifically enriched with O3FA.

## Resveratrol and O3FA Combined Effect

Many *in vivo* and *in vitro* studies testing the combined effects of O3FA and resveratrol have shown them to have synergistic effects on inflammation. For example, a recent study by Schwager et al. (68) showed that the LPS-stimulated peripheral leukocytes and IL-1-activated human chondrocytes treated only with resveratrol significantly reduced levels of prostaglandin 2 (PGE2), CCL5/RANTES, and CXCL8/IL-8, but increased IL-1β, IL-6, and IL-10, whereas O3FA treatment increased the levels of PGE2 and CXCL8/IL-8. Surprisingly, their combined administration exerted joint effects on CCL5/RANTES and IL-6 or CXCL8/IL-8 (68). Thus, O3FA and resveratrol act together on inflammatory mediators when dealing with acute and chronic inflammation of chondrocytes, but not on acute inflammation of peripheral leukocytes. In diseases where chronic inflammation occurs, such as osteoarthritis, O3FA and resveratrol may be beneficial. *In vitro* studies of chronic inflammation implementing long-term treatment of these two have shown a reduction in the production of inflammatory mediators. For example, bovine chondrocytes and cartilage explants have identified that long-term *in vitro* treatment of chondrocytes with conjugated linoleic acids or EPA mitigated the production of PGE_2_ and nitric oxide (69). Also, other studies reported reduced mRNA levels of matrix metalloproteinaises (MMPs), a disintegrin and metalloproteinase with thrombospondin motifs 4 (ADAMTS-4), and interleukins (68, 70). Thus, resveratrol and O3FA are able to reduce the inflammatory processes during chronic inflammation. But, in instances of acute inflammation, resveratrol acts to reduce early inflammatory events, whereas O3FA has the opposite effect and increases inflammatory events.

In an attempt to identify an effective therapy for cardiovascular patients, Hobbs et al. (71) did a pilot study with a multi-ingredient supplement (MIS) featuring blend of red yeast rice, bioflavonoids, polycosanol, and resveratrol along with a high-potency omega-3 polyunsaturated fatty acid in 19 patients with hypercholesterolemia (71). They found that the MIS supplement offers a promising result for cholesterol management by decreasing total cholesterol and LDL significantly. A separate review by the World Health Organization (72) found that the Mediterranean diet (MeDi) is a promising strategy to prevent diseases such as cerebro- and cardiovascular diseases, cancer, respiratory diseases, diabetes, and neurodegenerative diseases, thereby improving life quality (72). The two major components in MeDi in this study are long-chain O3FAs derived from fish and resveratrol from plant phenols. The MeDi benefits have been studied in many human epidemiological studies suffering from cancer, cardiovascular conditions (myocardial infarction, stroke, angina pectoris, coronary bypass, and coronary angioplasty), and neurodegenerative diseases such as Alzheimer’s disease (AD) (73–76). Also, a recent long-term randomized clinical trial (RCT) in 334 participants with high cardiovascular risk at a mean age of 67 years (PREDIMED study) reported positive effects of MeDi (77, 78).

From these studies, it is evident that the combinational therapy with O3FAs and resveratrol is more beneficial, and therefore, more studies should be directed to understand more insight on their mechanism of action at a molecular level.

## Conclusion

**Figure 1:**
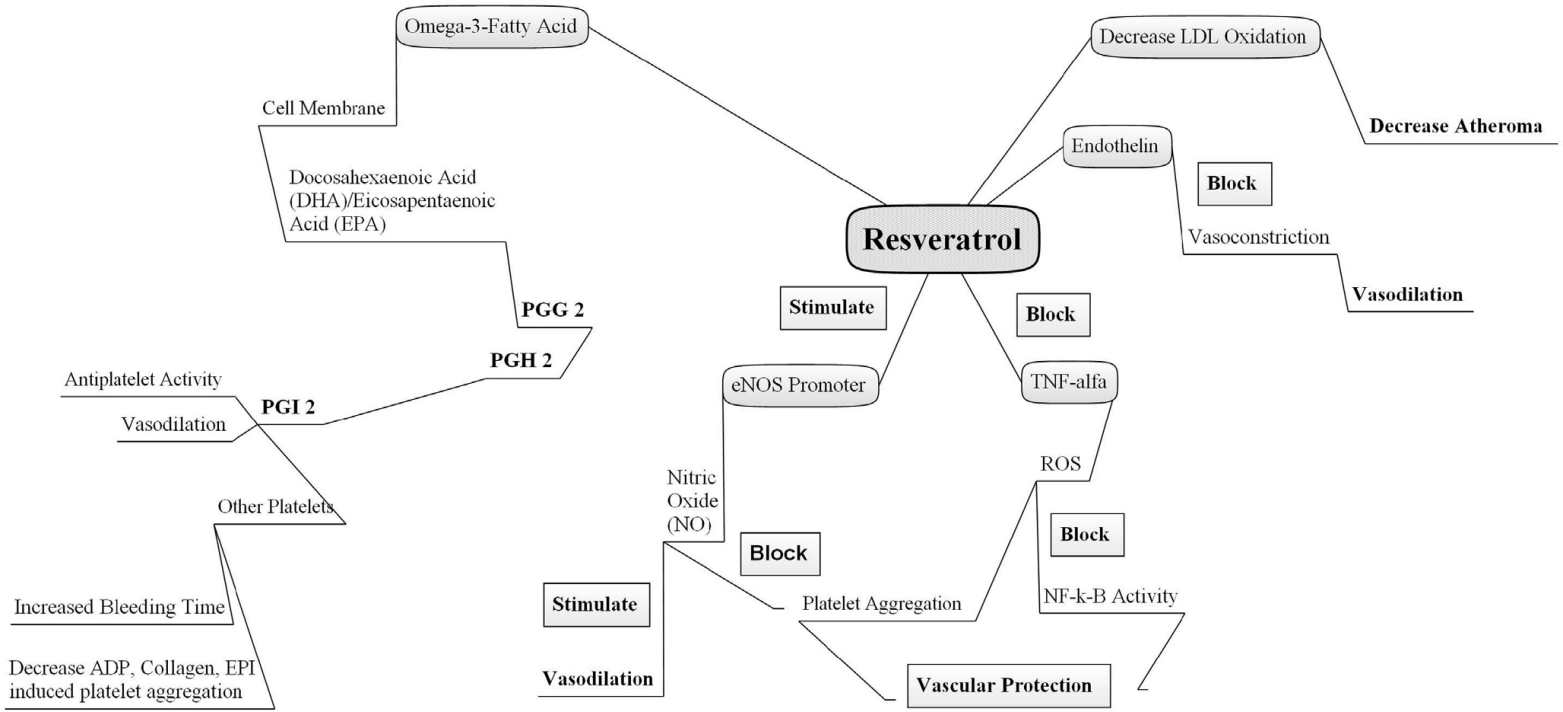
**Mechanisms of action of omega-3 fatty acid and resveratrol**. Two natural cardioprotective compounds act independently via different pathways combination of both may offer beneficial effects. PGG_2_, prostaglandin G_2_; PGH_2_, prostaglandin H_2_; PGI_2_, prostaglandin I_2_ or prostacyclin; eNOS, endothelial nitric oxide synthase; NO, nitric oxide, TNF-alpha, tissue necrotic factor alpha; ROS, reactive oxygen species; LDL, low density lipoprotein; ADP, adenosine diphosphate; NF-κB, nuclear factor kappa B.

Based on the existing literature discussed in this review, it is evident that resveratrol and O3FAs are reported to be significantly protective and play crucial roles in the prevention of not only various cardiovascular diseases but also a variety of other diseases, including Alzheimer disease, cancer, diabetes, and inflammation (Figure 1). Resveratrol and O3FAs, with their unique antioxidant properties and cardioprotective nature, are demonstrated to be highly functional and beneficial ingredients in various functional foods, although the number of studies in terms of dosage regimens and pharmacokinetics profiles of these ingredients is scanty and newer studies should be initiated to profile the same. The growing body of scientific literatures indicated the potential health benefits of these functional ingredients. However, new mechanistic studies should be designed to evaluate and identify the mechanisms behind the observed fascinating beneficial outcomes of resveratrol and O3FAs along with their combinational benefits.

## Conflict of Interest Statement

The authors declare that the research was conducted in the absence of any commercial or financial relationships that could be construed as a potential conflict of interest.
